# The efficiency of mandibular mini-implants in reducing adverse effects of class II elastics in adolescent female patients: a single blinded, randomized controlled trial

**DOI:** 10.1186/s40510-021-00368-2

**Published:** 2021-08-16

**Authors:** Mostafa M. El-Dawlatly, Mohamed A. Mabrouk, Amr ElDakroury, Yehya A. Mostafa

**Affiliations:** 1 Department of Orthodontics Faculty of Oral and Dental Medicine, Cairo University, Cairo, Egypt; 2 Department of Orthodontics, Future University in Egypt (FUE), Cairo, Egypt

**Keywords:** Class II treatment, Class II elastics, Mini-implants

## Abstract

**Background:**

Excessive proclination of lower incisors and other undesirable consequences usually result from the use of class II elastics during orthodontic treatment. The purpose of this study was to attempt to limit the adverse effects of class II elastics by the use of mini implants placed in the mandibular arch in adolescent class II female patients.

**Methods:**

The sample comprised 28 patients, (a mean age of 15.66 ± 2 years for intervention group and 15.1 ± 2.2 years for conventional group) with one-fourth or one-half unit class II canine relationship. The sample was divided into two equal groups. Randomization was carried out by a computer sequence generator with a 1:1 allocation ratio. In the intervention group, the mini implants were inserted between the lower second premolar and first molar, while the conventional group underwent regular class II elastics therapy. The active elastics treatment time was 8 months for both groups. Results were assessed by measurements from pre- and post-elastics lateral cephalometric radiographs.

**Results:**

The change in L1 inclination (0.97 ± 0.92°) and L1 AP position (0.31 ± 0.63 mm) did not show a statistically significant difference between the two groups, but a statistically significant difference was found in the U1 retroclination (5.23 ± 1.92°) and U1 distal movement (4.05 ± 1.4 mm) [P ˂ 0.001] and [P ˂ 0.05] respectively in favor of the intervention group.

**Conclusion:**

Mini-implants in conjunction with class II elastics had no skeletal effect, mainly dentoalveolar and it did not prevent the proclination of lower incisors. There was more distal movement in the upper incisors in the skeletal anchorage group which helped in enhancing the camouflaging of class II malocclusion.

**Trial registration:**

Trial registered “FUE.REC (10)/10-2018” at the FUE registration council for clinical trials/IOP Orthodontic Program October 2018.

## Introduction

Class II malocclusion is characterized by an incorrect relationship between the maxillary and mandibular arches due to skeletal or dental discrepancies or a combination of both. The overall global prevalence of this malocclusion was found to be 19.56% among adolescents in different populations, besides, class II division 1 was the most prevalent occlusal pattern [1, 2].

In growing patients having class II mandibular retrusion, functional appliances are commonly used for mandibular advancement based on the concept of growth modification [3]. In adult patients with class II malocclusion (full unit), camouflage protocols are usually used involving the extraction of maxillary first premolars. In patients having quarter or half unit molar and canine class 2 relation, non-extraction protocols usually apply by using class II elastics [4]. However, several problems appeared to compromise the desired outcomes of such treatment, such as the flaring of lower incisors, the over eruption of mandibular first molars, and the retroclination of upper incisors. These adverse effects can lead to an incomplete correction of the class II dental relationship [5].

Several attempts were proposed to counteract the unwanted dento-alveolar side effects of class II elastics. The use of cinch backs to prevent lower incisor proclination is the most widely used method. Some studies used skeletal anchorage in an attempt to limit the unwanted dental effects of fixed functional appliances. These studies found that anchorage using mini implants reduced the lower incisors proclination but they in turn increased the upper incisors retroclination and were not able to achieve significant skeletal mandibular growth [6, 7].

The use of mini implants was established to be well accepted by patients and providers, safe, and effective adjunct for complex orthodontic cases [8]. However, the use of mini-implants, for the purpose of reducing the proclination of the lower incisors, was restricted in the literature to the fixed functional appliances therapy. The current study attempted to use mini implants anchorage to reduce some of the unwanted dental effects accompanied by class II elastics therapy in a group of adolescent females. The null hypothesis of the current research was that there is no difference in the proclination of the lower incisors after the use of class II elastics whether mini-implants were used or not.

## Methods

Twenty-eight adolescent female patients (mean age, 15.66 ± 2 years for mini-implant group and 15.1 ± 2.2 years for conventional group) were randomized in a 1:1 ratio in 2 groups (14 patients in each group). Patients who showed up for orthodontic treatment with fixed appliances were recruited at the outpatient clinic of the Orthodontic Department in the University. The inclusion criteria were mild to moderate class II malocclusion (¼ to ½ unit canine relationship), no caries, missing teeth, periodontal disease, and adequate oral hygiene. Subjects were excluded if they were unwilling to be assigned to any of the approaches or had any abnormal oral or medical condition contraindicating orthodontic treatment. Consent was obtained from the patient’s parents, as they were adolescents, before their recruitment. Approval of the college ethics committee “FUE.ESTHECIS (10)/10-2018” was obtained before embarking on the treatment.

### Interventions

**Fig. 1:**
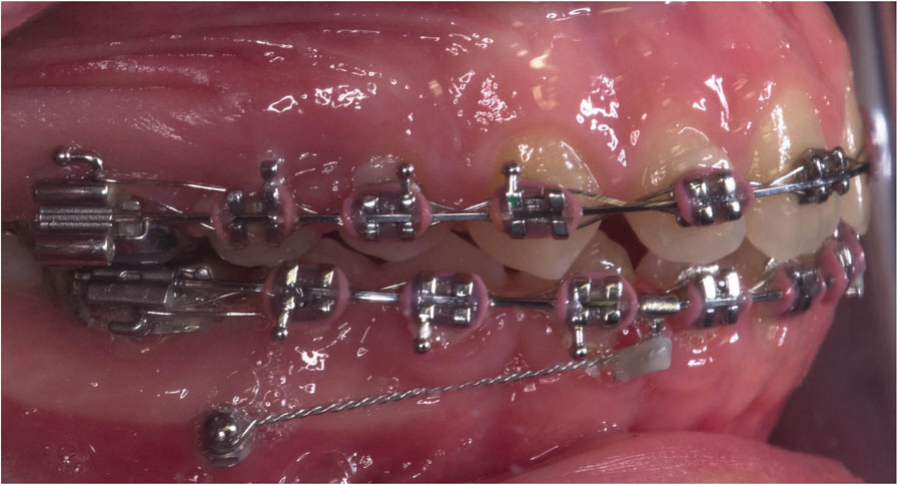
Intra-oral lateral view with mini-implants inserted between the lower second premolar and first molar, ligated to crimpable hook on the arch wire (mini- implant group)

**Fig. 2:**
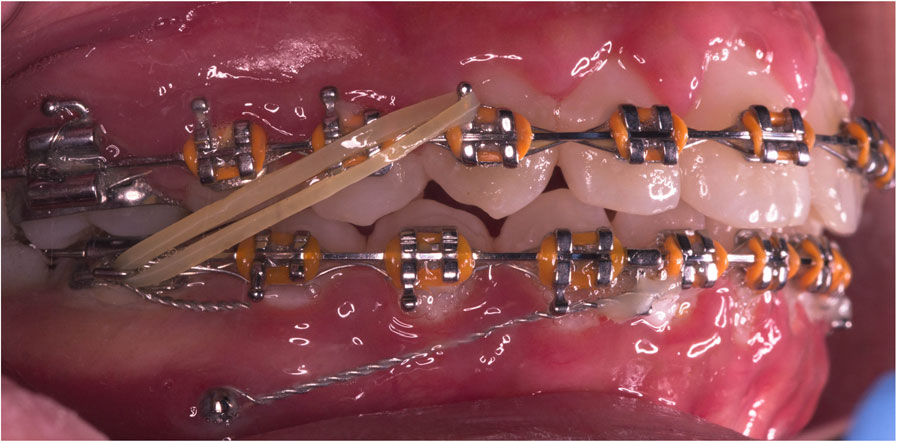
Intra-oral lateral view with class II elastics (mini-implant group)

The sample was divided randomly into two equal groups. The conventional group underwent regular treatment using class II elastics. While the mini-implant group comprised the use of class II elastics combined with mini implants (Figs. 1 and 2). The 1st molars were banded with molar bands. The rest of the teeth were bonded with Roth 0.022 slot sized brackets. Leveling and alignment was done by following the normal sequence of wires: 0.014 NiTi, 0.016 NiTi, 0.016 × 0.022 NiTi wires, 0.017 × 0.025NiTi wires, and 0.017 × 0.025 stainless steel (SS) wires, ligating the upper and lower arches from the first molar on one side to the first molar on the other side. Mini-implants were then inserted buccally in the inter-radicular bone between the lower 2nd premolar and 1st molar in both sides of the mandible. Short crimpable hooks were added on rectangular 0.017 × 0.025 stainless steel (SS) wire distal to both lower lateral incisors. 0.010 ligature wire was used for fixation of the mini-implants to the hooks. Maximum tightening of the ligature wire was done aiming to hinder the “mesial kick” produced by the class II elastics in the lower arch. The crimpable hooks and ligature were fixed in place using flowable composite (Figs. 1 and 2).

The force of the elastics was adjusted at 150 g per side. This was accomplished using a force gauge and adjusted by using the appropriate size of elastics tailored to every case individually. Patients were educated on how to use the class II elastics and were instructed to start using it daily (24 h per day). A timetable was given for each patient during the use of class II elastics in order to record the number of hours of wear per day.

**Fig. 3:**
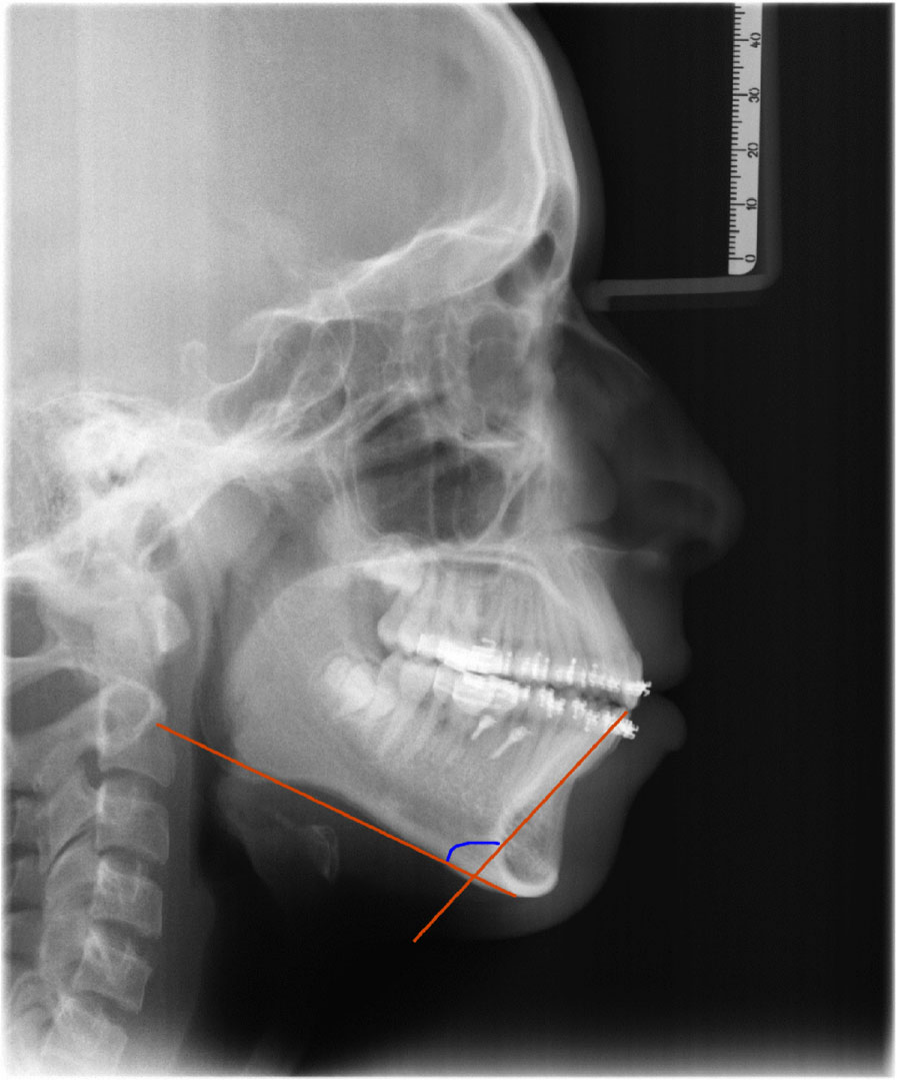
L1 mandibular plane angle, the primary outcome of the study

**Fig. 4:**
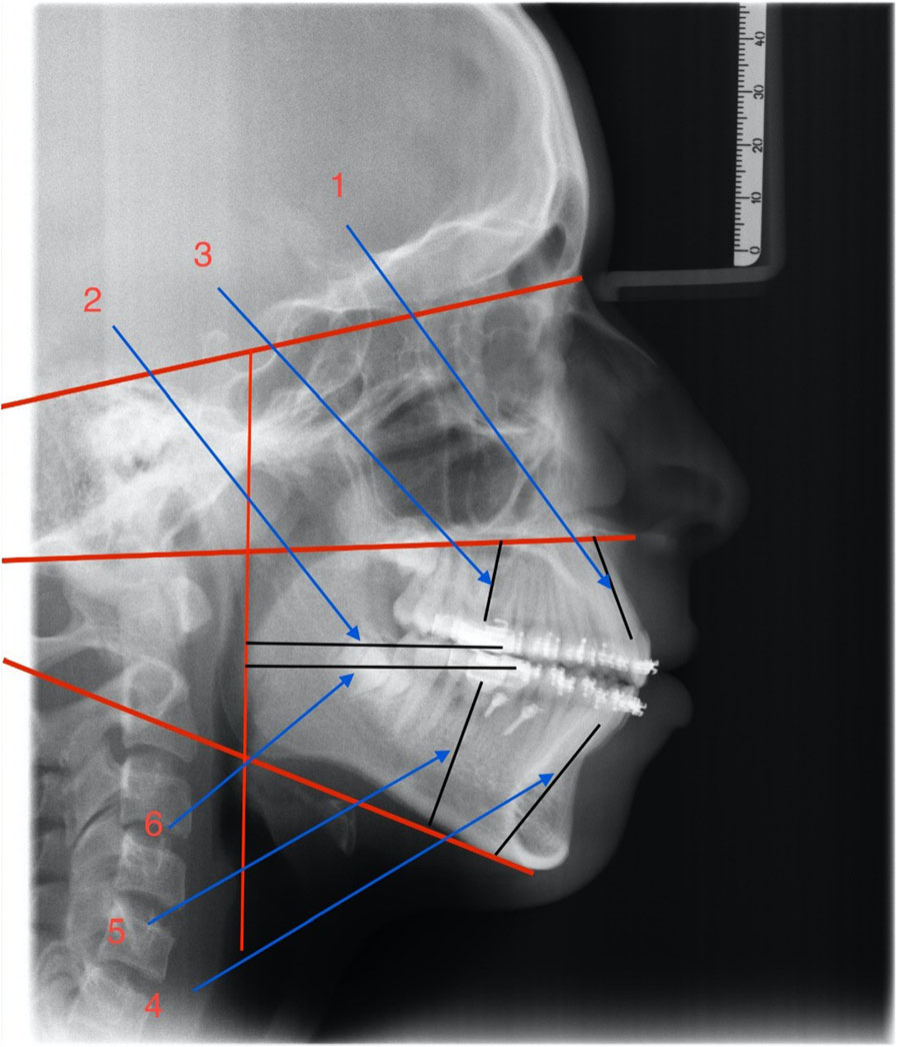
Linear measurements: (1) U1 vertical position (2) U6 AP position (3) U6 vertical position (4) L1 vertical position (5) L6 vertical position (6) L6 AP position

Patients were asked to attend for follow-up visits every 3 weeks for checking the progress and evaluation of the stability of mini-implants. Both groups placed the class II elastics for 8 months. Results were assessed by measurements from the lateral cephalometric radiograph (Table 1) (Figs. 3 and 4). Data collection of the outcome was done by importing pre and post class II elastics lateral cephalometric radiographs, into medical imaging software (Oris Ceph, Elite Computer, Vimodrone, Milano, Italy) (Table 1), (Figs.3 and 4).

**Table 1:** Definitions of the included measurements in the study

**Measurement**	**Definition**
MMP	The three-dimensional (3D) angle between the palatal line and the mandibular plane; maxillary-mandibular plane angle.
MP/SN	The 3D angle between the line S-N and the mandibular plane
Y axis angle	The 3D angle between y-axis and Frankfurt horizontal plane
Facial height ratio	The ratio of lower to total facial height
Lower facial height	The distance between the anterior nasal spine (ANS) and the Menton (Me)
SNA	The angle between the points S, N, and A
Effective maxillary length	The linear distance between the Condylion and A points
SNB	The angle between the points S, N, and B
effective mandibular length	The linear distance between the Condylion and the Gnathion points
	The angle between three landmarks: A, N, and B
SN/occlusal plane	The angle between the line passing from sella turcica to nasion and the occlusal plane
U1 AP position	The horizontal distance between the incisal edges of the upper central incisors and the frontal plane, as viewed from the sagittal view.
U1 vertical position	The linear distance from the midroot of the upper incisors to the FHP, as viewed from the sagittal view
U1 inclination	The angle formed between the frontal plane and the upper right and left central incisor long axes, as viewed from sagittal view
U6 AP position	The linear distance between the mesio-buccal cusp tip of UR6 and the vertical plane, as viewed from the sagittal view.
U6 vertical position	The linear distance between the furcation area of the upper right first molar to the FHP, as viewed from the sagittal view.
L1 inclination	The angle formed between the frontal plane and the lower incisors long axes, as viewed from the sagittal view
L1 AP position	The horizontal distance between the incisal edges of the lower incisors and the frontal plane, as viewed from the sagittal view.
L1 to NB line	The horizontal distance between the incisal edges of the lower incisors and the NB line, as viewed from the sagittal view
L1 vertical position	The linear distance from the midroot of the lower incisors to the mandibular plane, as viewed from the sagittal view
L6 vertical position	The linear distance from the furcation points of the lower right first molar to the mandibular plane, as viewed from the sagittal view
L6 AP position	The linear distance between the mesio-buccal cusp tip of lower left first molar and the vertical plane, as viewed from the sagittal view.

### Sample size calculation

Sample size calculation was done to detect a minimum clinically relevant difference in the lower incisors’ inclination scores (primary outcome), between the mini-implant and conventional groups based on a previous study by ELkordy et al. [8]. The difference in the means of the scores of the 2 groups was set at 3.5° with a standard deviation of 2.9°. The calculation indicated that for a study with a power of 80% and an alpha error of 0.05, it is required to have 12 participants per group. The number was raised to 14 to compensate for any expected drop-outs. The calculation was carried out using PS: Power and Sample Size calculator software Version 3.1.2 (Vanderbilt University, Nashville, TN, USA)

### Randomization

Patients were randomly allocated to group A (mini-implant) or B (conventional) using random sequence generated at random.org. Simple randomization was done with allocation ratio 1:1. The randomization was done before the start of treatment. Each number from the generated sequence (from 1 to 28) was put in an opaque sealed envelope and all envelopes were placed in a closed box. After completion of the leveling and alignment, each patient was requested to choose one envelope. According to the number in the envelope, the patient was then allocated into one of the two groups. The randomization and allocation steps were carried out by the clinic instructor who was not a part of the study.

### Blinding

Blinding was not applicable for the operators during the insertion of the appliance and the follow-up visits. The clinic instructor was responsible for assigning subjects to mini-implant or conventional groups according to the concealed allocation. Appliance activation at follow-up visits, dental impressions, and acquisition of dental casts were the responsibility of the principal investigator. It was a single-blinded study; only the outcome assessors were blind. The patient names were removed from pre- and post-treatment lateral cephalometric radiographs and study models. Then, two assessors carried on, blindly and independently, the measurements and analysis of the study.

### Statistical analysis

Statistical analysis was divided in 3 sections:

1. Comparing the skeletal and dental changes between the conventional and mini-implant groups (primary outcome).
2. Analyzing pre and post, skeletal, and dental changes in conventional group (secondary outcome).
3. Analyzing pre and post, skeletal, and dental changes in mini-implant group (secondary outcome).

All data were collected, tabulated, and subjected to statistical analysis. Statistical analysis was performed by SPSS in general (version 16), while Microsoft office Excel was used for data handling and graphical presentation.

Quantitative variables were described by the mean, standard deviation (SD), the range (minimum–maximum), standard error (SE), and 95% confidence interval of the mean.

Qualitative categorical variables were described by proportions and Percentages.

Shapiro-Wilk test of normality was used to test normality hypothesis of all quantitative variables for further choice of appropriate parametric and non-parametric tests. Mainly, the variables were found to be normally distributed allowing the use of parametric tests. Paired t test was applied for comparing the changes pre- and post-treatment within each group and Independent samples t test for comparing the differences (post–pre) between the two groups.

For reliability analysis of inter- and intra-observer of all measured variables, Dahlberg error (DE) and relative Dahlberg error (RDE) were used together with concordance correlation coefficients (CCC) including its 95% confidence limits.

Significance level was considered at *P* < 0.05 (S); while for *P* < 0.01 was considered highly significant (HS). Two-tailed tests were assumed throughout the analysis for all statistical tests.

## Results

The total sample comprised 28 adolescent female patients (divided into 2 groups (14 per each group). The mean ages were 15.66 ± 2 years for the mini-implant group and 15.1 ± 2.2 years for the conventional group. There were no drop-outs, where all the patients completed the full length of treatment.

The treatment time (including leveling and alignment and elastic wear period excluding the finishing stage) was about 14.75 ± 1.8 months for the mini-implant group and 15.12 ± 1.67 for the conventional group. The difference in the time interval of the use of the elastics was insignificant between the two groups (*P* > 0.05) (mini-implant group 8 ± 0.93 months and control group 7.47 ± 0.52 months)

There was no significant difference in the degree of compliance and adherence to elastics wear between the two groups. (*P* > 0.05). Where the mean hours the elastics were worn in the mini-implant group was 19.13 ± 1.68 h/day, while it was 18.7 ± 1.22 for the control group.

Baseline analysis was done before the trial by comparing conventional group and mini-implant group pre-treatment measurements SNB, ANB, and MMP, Effective mandibular length and L1 inclination. There was no statistically significant difference between the two groups. Excellent inter-observer and intra-observer reliability were detected, since RDE did not exceed 10% and CCC values were recorded between 0.991 and 1.

Statistical results were divided as follows:

## Analyzing pre and post, skeletal and dental changes in group: (Table 2)

The skeletal measurements that showed statistically significant change:

- Increase in the MP/SN, Y-axis angle, lower facial height and in the clock-wise rotation of the occlusal plane (SN/occlusal plane).

**Table 2:** Paired t test for comparing the dental and skeletal changes after class II elastics phase for the conventional group

Variable				Paired differences			t	*P* value	
		Mean	SD	Mean	SD	SEM			
**SNA**	**Pre**	82.08	2.97	− 0.33	2.23	0.64	− 0.52	0.61474	***P*> 0.05 NS**
	**Post**	81.75	3.19						
**SNB**	**Pre**	76.25	3.22	0.17	2.79	0.81	0.21	0.83988	***P*> 0.05 NS**
	**Post**	76.42	3.68						
**ANB**	**Pre**	5.83	1.11	− 0.50	1.57	0.45	− 1.11	0.29252	***P*> 0.05 NS**
	**Post**	5.33	1.72						
**MMP**	**Pre**	28.08	3.45	0.42	2.02	0.58	0.71	0.48993	***P*> 0.05 NS**
	**Post**	28.50	3.18						
**MP/SN**	**Pre**	36.08	4.58	1.38	2.23	0.64	2.14	0.05573	***P*≈ 0.05 Almost S**
	**Post**	37.46	5.27						
**Y axis angle**	**Pre**	57.20	3.70	2.30	4.67	1.35	5.62	0.00015	***P*< 0.001 HS**
	**Post**	59.50	3.75						
**Lower facial height**	**Pre**	63.70	5.47	2.30	4.91	1.42	3.36	0.00637	***P*< 0.01 HS**
	**Post**	66.00	4.47						
**Effective maxillary length**	**Pre**	83.17	4.20	0.75	5.28	1.52	0.49	0.63212	***P*> 0.05 NS**
	**Post**	83.92	4.68						
**Effective mandibular length**	**Pre**	108.50	5.52	2.00	8.34	2.41	0.83	0.42406	***P*> 0.05 NS**
	**Post**	110.50	6.08						
**SN/occlusal plane**	**Pre**	16.66	3.44	4.30	2.65	0.77	5.62	0.00016	***P*< 0.001 HS**
	**Post**	20.96	3.69						
**U1 vertical position**	**Pre**	20.92	3.17	1.67	3.39	0.98	1.70	0.11628	***P*> 0.05 NS**
	**Post**	22.58	2.81						
**U1 AP position**	**Pre**	7.30	3.06	− 0.30	3.82	1.10	− 0.27	0.79047	***P*> 0.05 NS**
	**Post**	7.00	1.76						
**U1 inclination**	**Pre**	113.00	4.13	− 3.33	1.61	0.47	3.33	0.00002	***P*< 0.001 HS**
	**Post**	109.67	4.42						
**U6 AP position**	**Pre**	42.17	2.92	− 2.04	5.91	1.71	2.04	0.25659	***P*> 0.05 NS**
	**Post**	40.13	4.89						
**U6 vertical position**	**Pre**	16.33	2.53	0.08	2.16	0.62	0.13	0.89619	***P*> 0.05 NS**
	**Post**	16.42	1.94						
**L1 inclination**	**Pre**	100.00	6.09	5.42	2.81	0.81	6.68	0.00003	***P*< 0.001 HS**
	**Post**	105.42	5.12						
**L1 AP position**	**Pre**	5.08	1.62	1.33	1.44	0.41	3.22	0.00819	***P*< 0.01 HS**
	**Post**	6.42	1.62						
**L1 vertical position**	**Pre**	30.75	4.77	2.42	4.70	1.36	1.78	0.10243	***P*> 0.05 NS**
	**Post**	33.17	3.93						
**L6 vertical**	**Pre**	23.54	2.68	1.21	0.99	0.29	− 1.21	0.00139	***P*< 0.001 HS**
	**Post**	24.75	2.83						
**L6 AP position**	**Pre**	42.08	3.23	0.92	6.35	1.83	0.50	0.62664	***P*> 0.05 NS**
	**Post**	43.00	5.77						

The dental measurements that showed statistically significant change:

- Increase in the proclination of L1, L1 AP position (anterior movement), L6 vertical position (extrusion), and the retroclination of U1.

1. Analyzing pre and post, skeletal, and dental changes in mini-implant group: (Table 3).

**Table 3:** Paired t test for comparing the dental and skeletal changes after class II elastics phase for the mini-implant group

Variable				Paired differences			t	*P* value	
		Mean	SD	Mean	SD	SEM			
**SNA**	**Pre**	82.78	3.00	− 1.12	2.50	0.72	− 1.55	0.14986	***P*> 0.05 NS**
	**Post**	81.67	3.42						
**SNB**	**Pre**	76.54	2.79	− 0.79	2.39	0.69	− 1.15	0.27516	***P*> 0.05 NS**
	**Post**	75.75	3.02						
**ANB**	**Pre**	6.20	1.47	− 0.37	0.79	0.23	− 1.62	0.13415	***P*> 0.05 NS**
	**Post**	5.83	1.11						
**MMP**	**Pre**	29.85	3.74	0.32	2.83	0.82	0.39	0.70541	***P*> 0.05 NS**
	**Post**	30.17	3.49						
**MP/SN**	**Pre**	38.68	4.67	0.37	2.13	0.62	0.60	0.56311	***P*> 0.05 NS**
	**Post**	39.04	4.50						
**Y axis angle**	**Pre**	56.85	3.35	2.15	1.32	0.38	5.62	0.00015	***P*< 0.001 HS**
	**Post**	59.00	2.98						
**Lower facial height**	**Pre**	62.53	3.60	2.13	2.20	0.64	3.36	0.00637	***P*< 0.01 HS**
	**Post**	64.67	4.64						
**Effective maxillary length**	**Pre**	84.13	4.57	0.70	3.00	0.87	0.81	0.43606	***P*> 0.05 NS**
	**Post**	84.83	4.32						
**Effective mandibular length**	**Pre**	106.37	5.26	1.97	4.31	1.24	1.58	0.14184	***P*> 0.05 NS**
	**Post**	108.33	5.65						
**SN/occlusal plane**	**Pre**	16.54	3.52	4.12	2.18	0.63	6.55	0.00004	***P*< 0.001 HS**
	**Post**	20.66	4.11						
**U1 vertical position**	**Pre**	20.73	2.89	1.06	2.31	0.67	1.59	0.14071	***P*> 0.05 NS**
	**Post**	21.79	1.95						
**U1 AP position**	**Pre**	13.18	3.00	− 4.35	2.99	0.86	− 5.04	0.00038	***P*< 0.001 HS**
	**Post**	8.83	2.48						
**U1 inclination**	**Pre**	115.94	2.04	− 8.57	6.45	1.86	− 4.60	0.00076	***P*< 0.001 HS**
	**Post**	107.38	5.48						
**U6 AP position**	**Pre**	44.17	6.10	− 3.29	6.38	1.84	− 1.79	0.10161	***P*> 0.05 NS**
	**Post**	40.88	4.86						
**U6 vertical position**	**Pre**	15.13	2.11	0.58	0.91	0.26	2.22	0.04819	***P*< 0.05 S**
	**Post**	15.71	2.08						
**L1 inclination**	**Pre**	99.88	4.80	4.45	1.47	0.42	10.52	0.00000	***P*< 0.001 HS**
	**Post**	104.33	4.12						
**L1 AP position**	**Pre**	4.78	1.19	1.03	1.63	0.47	2.18	0.05173	***P*≈ 0.05 Almost S**
	**Post**	5.81	1.59						
**L1 vertical position**	**Pre**	31.68	2.36	0.87	2.02	0.58	1.49	0.16480	***P*> 0.05 NS**
	**Post**	32.54	3.20						
**L6 vertical**	**Pre**	22.14	2.17	1.19	0.84	0.24	4.93	0.00045	***P*< 0.001 HS**
	**Post**	23.33	1.83						
**L6 AP position**	**Pre**	42.18	4.47	2.98	3.82	1.10	2.71	0.02043	***P*< 0.05 S**
	**Post**	45.17	3.69						

The skeletal measurements that showed statistically significant change:

- Increase in the Y-axis angle, lower facial height, and the clock-wise rotation of occlusal plane (SN/occlusal plane).

The dental measurements that showed statistically significant change:

- Decrease in the U1 AP position, U1 inclination (retroclination)
- Increase in the proclination of L1, L1 AP position (anterior movement), L6 vertical position (extrusion), L6 AP position (mesial movement).

2. Comparing the skeletal and dental changes between the conventional and mini-implant groups: (Table 4).

**Table 4:** Independent samples T test for comparing the differences in the dental and skeletal changes after class II elastics phase for between the 2 groups

Variable	Group			Differences	95% Confidence interval of the difference			t	*P* value	
		Mean	SD	Mean	SD	Lower	Upper			
**SNA**	**C**	− 0.33	2.23	0.78	0.97	− 1.22	2.79	0.81	0.42642	***P*> 0.05 NS**
	**I**	− 1.12	2.50							
**SNB**	**C**	0.17	2.79	0.96	1.06	− 1.24	3.16	0.90	0.37587	***P*> 0.05 NS**
	**I**	− 0.79	2.39							
**ANB**	**C**	− 0.50	1.57	− 0.13	0.51	− 1.18	0.92	− 0.26	0.79458	***P*> 0.05 NS**
	**I**	− 0.37	0.79							
**MMP**	**C**	0.42	2.02	0.10	1.00	− 1.98	2.18	0.10	0.92150	***P*> 0.05 NS**
	**I**	0.32	2.83							
**MP/SN**	**C**	1.38	2.23	1.01	0.89	− 0.84	2.85	1.13	0.26928	***P*> 0.05 NS**
	**I**	0.37	2.13							
**Y axis angle**	**C**	2.30	4.67	0.15	1.40	− 2.76	3.06	0.11	0.91572	***P*> 0.05 NS**
	**I**	2.15	1.32	0.15						
**Lower facial height**	**C**	2.30	4.91	0.17	1.55	− 3.05	3.39	0.11	0.91545	***P*> 0.05 NS**
	**I**	2.13	2.20	0.17	1.55	− 3.14	3.47	0.11		
**Effective maxillary length**	**C**	0.75	5.28	0.05	1.75	− 3.58	3.68	0.03	0.97749	***P*> 0.05 NS**
	**I**	0.70	3.00							
**Effective mandibular length**	**C**	2.00	8.34	0.03	2.71	− 5.59	5.65	0.01	0.99030	***P*> 0.05 NS**
	**I**	1.97	4.31							
**SN/occlusal plane**	**C**	4.30	2.65	0.18	0.99	− 1.87	2.24	0.19	0.85482	***P*> 0.05 NS**
	**I**	4.12	2.18							
**U1 vertical position**	**C**	1.67	3.39	0.61	1.18	− 1.85	3.06	0.51	0.61232	***P*> 0.05 NS**
	**I**	1.06	2.31							
**U1 AP position**	**C**	− 0.30	3.82	4.05	1.40	1.15	6.95	2.89	0.00842	***P*< 0.01 HS**
	**I**	− 4.35	2.99							
**U1 inclination**	**C**	− 3.33	1.61	5.23	1.92	1.26	9.21	2.73	0.01227	***P*< 0.05 S**
	**I**	− 8.57	6.45							
**U6 AP position**	**C**	− 2.04	5.91	1.25	2.51	− 3.96	6.46	0.50	0.62360	***P*> 0.05 NS**
	**I**	− 3.29	6.38							
**U6 vertical position**	**C**	0.08	2.16	− 0.50	0.68	− 1.90	0.90	− 0.74	0.46804	***P*> 0.05 NS**
	**I**	0.58	0.91							
**L1 inclination**	**C**	5.42	2.81	0.97	0.92	− 0.93	2.86	1.06	0.30225	***P*> 0.05 NS**
	**I**	4.45	1.47			− 0.97	2.90	1.06		
**L1 AP position**	**C**	1.33	1.44	0.31	0.63	− 0.99	1.61	0.49	0.62748	***P*> 0.05 NS**
	**I**	1.03	1.63							
**L1 vertical position**	**C**	2.42	4.70	1.55	1.48	− 1.51	4.61	1.05	0.30515	***P*> 0.05 NS**
	**I**	0.87	2.02							
**L6 vertical**	**C**	1.21	0.99	0.02	0.37	− 0.76	0.79	0.04	0.96483	***P*> 0.05 NS**
	**I**	1.19	0.84							
**L6 AP position**	**C**	0.92	6.35	− 2.07	2.14	− 6.50	2.37	− 0.97	0.34422	***P*> 0.05 NS**
	**I**	2.98	3.82							

*C* conventional group, *I* mini-implant group

Only two dental measurements showed statistically significant difference. Those were the lingual movement of the upper incisors U1 AP position and the retroclination of the upper incisors U1 inclination (Table 4). All the other skeletal and dental measurements showed no statistical significance.

The overjet was reduced significantly in both mini-implant and control groups (*P* < 0.001); with a mean of − 4.2 ± 0.71 in the mini-implant group and − 3.81 ± 0.8 in the conventional group, while the difference between them was not significant 0.39 ± 0.31 (*P* > 0.05). Harms: no serious adverse effects were observed other than gingivitis associated with plaque accumulation.

**Fig. 5:**
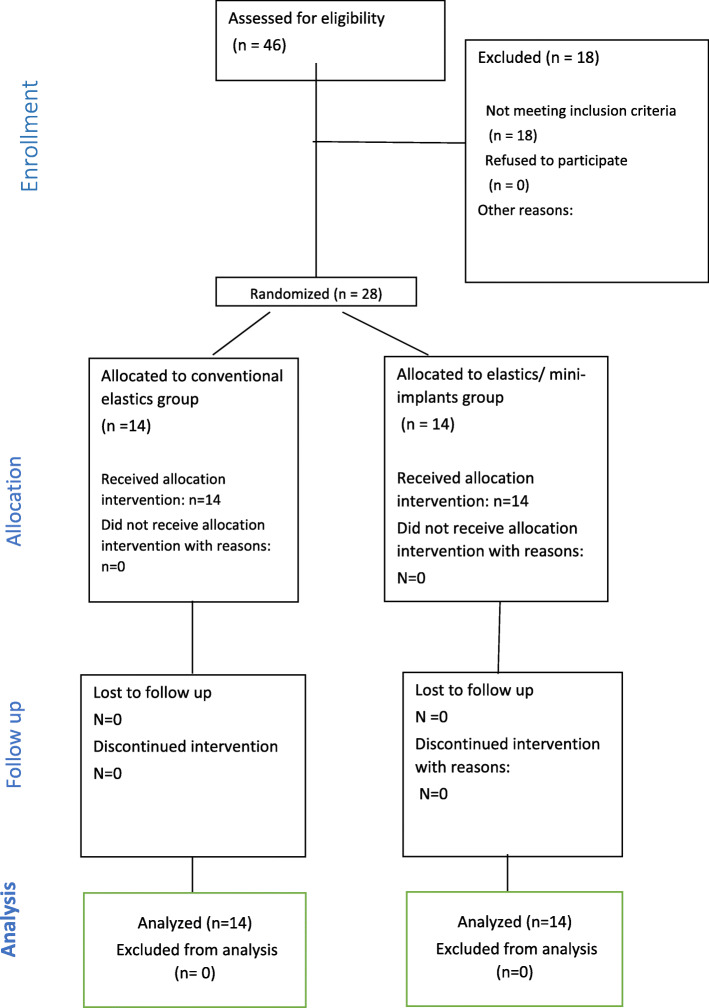
Consort flow diagram

**Fig. 6:**
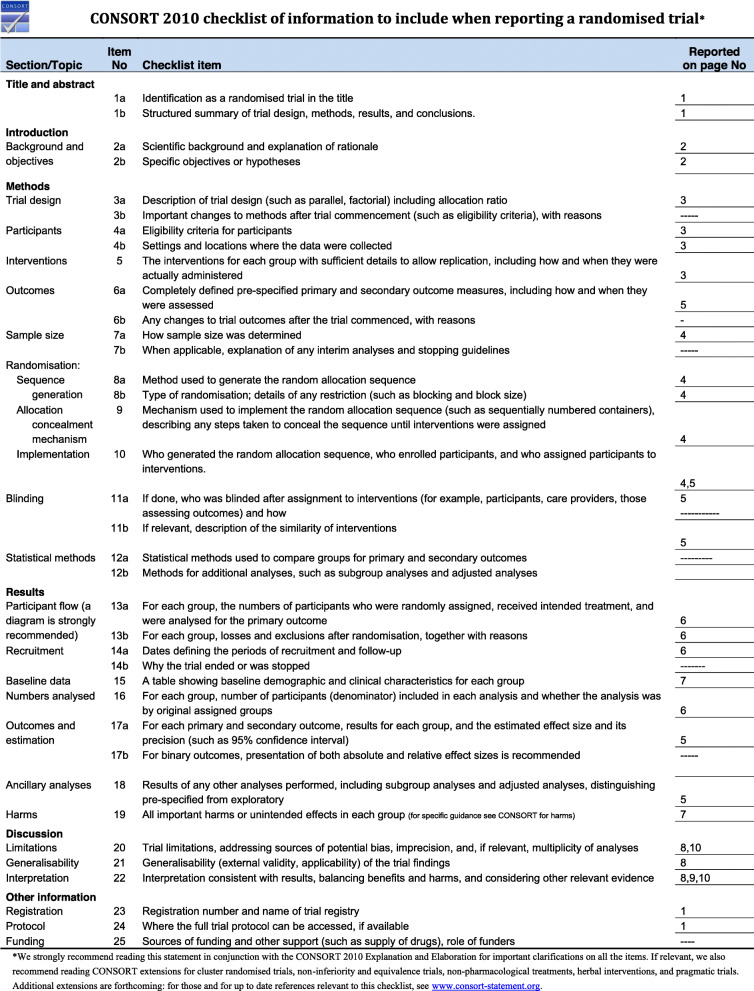
consort check list

A consort flow diagram (Fig. 5) together with a consort check list (Fig. 6) was made to describe the steps of the current randomized controlled trial.

## Discussion

Class II elastics could be used to camouflage mild skeletal class II or to treat mild to moderate dental class II malocclusions. The use of class II elastics could be extended to anchorage reinforcement, bite opening (in cases having class II division2) [9], midline deviation correction. The adverse effects of using class II elastics comprise both vertical and horizontal force vectors [9]. This vertical force extrudes the maxillary incisors and mandibular molars and can lead to clock-wise rotation of the occlusal plane. The horizontal vector of force has been shown to cause the mandibular first molars to rotate or tip mesially, procline the mandibular anterior teeth, and displace the entire lower dental arch anteriorly [10].

Many studies investigated the effect of class II elastics and compared its effects versus those of fixed functional appliances, but none of them used skeletal anchorage to prevent or counteract the drawbacks of class II elastics [11]. Accordingly, we attempted to use mini implants anchorage aiming to reduce the lower incisors proclination and decrease the extrusion of lower first molars accompanied by class II elastics in non-extraction cases.

The adolescent female patients included in the sample were in their early post-pubertal age. Only female patients were recruited, aiming to eliminate the potential residual growth differences between males and females in this age group. Representing a limitation, this unfortunately restricted the generalization of the results of the current study and limited it to the female gender.

Moreover, the results should be interpreted with caution due to the limited sample. In addition, the current study tested a new genuine technique; accordingly, the sample size calculation was based on a study that used the same skeletal anchorage protocol but on a fixed functional appliance [8] instead of class II elastics.

Mini-implants were placed between the lower second premolar and first molar as it was stated by Fayed et al. [12] that this site is considered one of the optimal sites for placing mini-implants in the lower arch. Eight-millimeter-long mini-implant was chosen as it was reported that shorter mini-implant will not be stable enough and longer mini-implants risks injury to the inferior alveolar nerve [13].

In the current study, lateral cephalograms were taken two times; before the start of class II elastics therapy and just after the end of the elastics phase for each patient. However, the cephalometric radiograph has the limitation of reduced validity and/or reproducibility of its landmarks; it remains the radiographic tool of choice for the sake of avoiding the high dose of the 3D cone beam CT. The acquisition of cephalometry at the start and end of treatment was a must in order to distinguish the results of elastics therapy excluding the incisors proclination effect of the leveling and alignment phase. This was compensated by avoiding the acquisition of a post-treatment cephalometry.

In the conventional group, the results showed that class II elastics were effective in correcting class II malocclusions, and their effects were mainly dentoalveolar, including lingual tipping, retrusion, and extrusion of the maxillary incisors; labial tipping of the mandibular incisors and mesialization and extrusion of the mandibular molars. Also, there was a significant clockwise rotation in the mandibular plane angle and occlusal plane. These results were in agreement with Janson et al. [11] when they evaluated the effects of class II elastics in class II malocclusion treatment in their systematic review.

In the skeletal anchorage group, the lower molars moved mesially significantly, and this revealed total movement of the whole mandibular dentition to a more forward position. These results were in agreement with those of Janson et al. [11] as they reported that class II elastics enhanced the forward movement of the mandibular first molars, moving 1.2 mm mesial, and lead to proclination of the mandibular incisors.

In the skeletal anchorage group, the use of mini-implants did not prevent the proclination of the lower incisors as was shown in the lower incisors proclination (4.45 ± 1.47°) and lower incisors anteroposterior position (1.03 ± 1.63 mm). The upper incisors were retroclined (− 8.57 ± 6.45°) and moved distally significantly (− 4.35 ± 2.99 mm). There was a significant increase in the retroclination (5.23 ± 1.92°) and distal movement (4.05 ± 1.4 mm) of the upper incisors. The distal movement in the upper incisors helped in a more pronounced camouflaging effect of the class II malocclusion in the mini-implant group [4]. The significant retroclination of the upper incisors lead to the increase of the incisal display which is beneficial to cases having decreased incisal show at rest and on smiling; this was confirmed by Armando Saga et al. [14] who found that retroclined maxillary incisors that occur after orthodontic anterior retraction without torque control tend to increase the incisor exposition and worsen an existing gummy smile. On the other hand, this will lead to further deepening of the bite [10, 1].

The significant proclination of lower incisors in both groups may be due to the degree of play between the wire and the brackets despite using 0.017 × 0.025 stainless steel arch wires which were heavier than the wires used in almost all articles investigating class II elastic wear. Janson et al. [11] stated in their systematic review that the current literature suggests using light forces (2.6 oz) obtained with 3/16 diameter elastics and a rectangular 0.016 × 0.022 stainless steel arch-wires. The use of ligature wires as a way of attachment between the mini-implant and the arch wire could have shared in the incapability of the current technique to reduce the flaring of the lower incisors. The use of other active methods of attaching the mini-implant, like elastic chains, in further studies might have a better impact on reducing the incisors proclination. Also, the use of 0.019 × 0.025 stainless steel arch wires could be tested in further studies and compared to other lower dimensional arch wires to reach a conclusive evidence in terms of the effect of the wire dimensions on class II elastics outcomes.

Other studies used the mini-implant to limit the proclination of lower incisors in conjunction with fixed functional appliances. They found reduction in their proclination in the mini-implant group which was not found in the present study [15, 16]. This could be attributed to the use of a different type of appliance or to the nature of force applied on the lower incisors, where the force delivered by the class II elastics has a more jiggling nature than that delivered by the fixed functional appliances.

Extrusion of lower first molar and upper anterior teeth with clockwise rotation of the mandible and occlusal plane were noticed in both groups. This proved that the use of skeletal anchorage in the mandibular arch could not prevent the adverse effects produced by the vertical force component of elastics attachment points. These findings agree with Janson et al. [4] study, reporting insignificant positional changes of teeth at the end of comprehensive class II elastic treatment but there was a significant increase in the vertical measurements.

There were no statistically significant differences in the skeletal variables in the skeletal anchorage group like SNA (− 1.12 ± 2.5°), SNB (0.79 ± 2.39°), and ANB (− 0.37 ± 0.79°); this agreed with Janson et al. [11] as they stated that class II elastics effect was about 18.9% skeletal and 71.1% dentoalveolar and the significant retroclination of the maxillary teeth worked against the mandibular advancement. Moreover, the mean age of the patients in the mini-implant group was 15.66 ± 2 years and 15.1 ± 2.2 years for the conventional group. Accordingly, most of the craniofacial growth had ended at the start of treatment, thus, limiting the potential for mandibular growth enhancement.

Very good adherence to the elastics was found in this study with an average of 19 h per day wearing time. No patients reported any severe pain related to elastics wear or mini-implants. The results of this study concluded that, mini-implants placed in the mandibular inter-radicular bone in conjunction with class II elastics had no skeletal effect. The dentoalveolar effect predominated and the current technique did not prevent the excessive proclination of lower incisors. It had some little positive effects, as it enhanced more mesial movement of mandibular dentition.

## Conclusion

- Mini-implants placed in the mandibular interradicular bone in conjunction with Class II elastics had no significant skeletal effects, only dentoalveolar movements resulted.
- Skeletal anchorage didn’t prevent the proclination of lower incisors, the extrusion of the lower buccal segment, or the clockwise rotation of the mandibular and occlusal planes.
- Skeletal anchorage increased the retroclination of the upper incisors and the distal movement of the upper incisors in the skeletal anchorage group which helped in enhancing the camouflaging effect of Class II malocclusion.

## Data Availability

The datasets used and analyzed during the current study are available from the corresponding author on reasonable request.

## Abbreviations

SS: Stainless steel
NiTi: Nickel titanium
